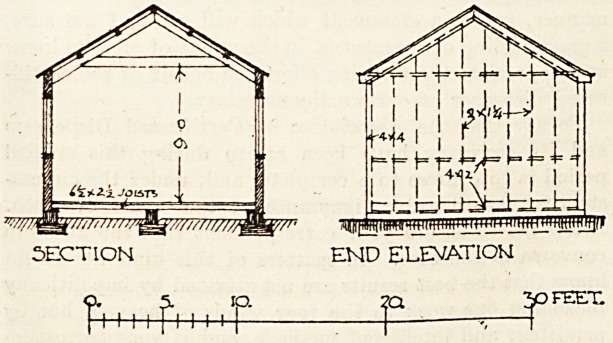# Our Bureau of Information

**Published:** 1911-11-18

**Authors:** 


### OUR BUREAU OF INFORMATION.

### Work for Consumptives in Sanatoria.

WISHING to make the Bureau of great practical use we are holding over other matter in order to deal this week with a question affecting sufferers from tuberculosis. In our issue of November 4 we published an inquiry from "Forward," asking for information about a Sanatorium Joiners' Shop for the use of its patients.

We have the pleasure to give this week the particulars asked for, including the style of building suitable for working in, in all weathers, with particulars as to cost and equipment. We invite other inquiries of this practical description from our readers.

JOINERS' WORKSHOP FOR CONVALESCENT PATIENTS.

The joiners' workshop illustrated below, to be used in all weathers by convalescent patients, was erected at a

A JOINERS WORKSHOP.

PLAN

FRONT ELEVATION

sanatorium and equipped complete at a cost of fifty pounds.

It consists of a shed twenty-four feet long and fourteen feet wide, with a height of nine feet to the roof beams. The roof is of 24 gauge corrugated iron sheets laid on

SECTION

END ELEVATION

4 by 2 purlins which are supported on 5 by 2 spars and collars, forming principals (see plan). The floor joists are 6½ by 2½, resting on wallplates on the brickwork base. The vertical corner-posts are 4 by 4 with 4 by 2 sills and posts, and the filling studs to carry corrugated iron are 2 by 1¼. All the windows are hinged to open; the door is of the simplest and cheapest form, known as "ledged and braced." A heating stove is placed at one end with its smoke flue of iron carried through the roof.

The cost of the corrugated iron was £7 6s. 8d.; five windows cost £3; the flooring £2 12s.; floor joists £2 9s. In this case a second-hand bench and set of tools were purchased at a cost of £16 10s; but if a regular workman was employed this would save some £15 on the outlay, as he provides his own tools. The cost of labour on this particular building amounted to £12, two men being employed for four weeks.

Though the building is not a beautiful one, it is roomy and well adapted for its purpose, and could no doubt be placed where it would not form an obtrusive feature in the landscape.

BROMPTON HOSPITAL SANATORIUM AND CONVALESCENT HOME, FRIMLEY.

DR. MARCUS PATERSON writes: We have in use a carpenters' shop such as is suggested. It is made with a completely open front; is 10 ft. deep, 11 ft. high in front, and 8 ft. at the back. A shed of these dimensions and about 15 ft. long could be erected for about £20. This will include a concrete floor and a galvanised iron roof. As regards equipment, £5 will provide a bench and necessary tools, but to do any quantity of work would probably mean a further expenditure of £15.

### Wanted Experienced Honorary Correspondents.

We want at once a correspondent at Oxford, Southbourne, Bedford, Malvern Link, Norwich, Clacton, Maidenhead, and Seaford. "Rochester" is thanked for his offer and testimonials, which have been returned through the post. "Felixstowe" is thanked, but must comply with the conditions printed last week. "Ledbury" is thanked.

### How to Make the Bureau a Success.

Everyone who uses or wishes to help our Bureau of Information must be kind enough to observe the following rules:

(1) Postal replies cannot be given. Exception will only be made in very special cases at the Editor's discretion.

(2) Queries or answers relating to different cases must be written, or preferably typed, on separate sheets of paper, and the writing must never be crossed.

(3) Questions and replies received not later than Wednesday will, as far as possible, be dealt with in our issue of the following Saturday week.

(4) Letters must have attached the name and address of the sender, with a pseudonym for publication if preferred.

(5) *All communications* for this department *must be accompanied by the coupon* to be found at the bottom of the third page of the cover on the inside, and should be addressed to the Editor of the Bureau of Information, c/o THE HOSPITAL, 29 Southampton Street, Strand, London, W.C.

*Notice.*—We invite workers of special experience and knowledge to offer help in replying to questions relating to their special department.

### Needs and Helpers.

SEVERAL correspondents are thanked and will be dealt with next week.

## Figures and Tables

**Figure f1:**
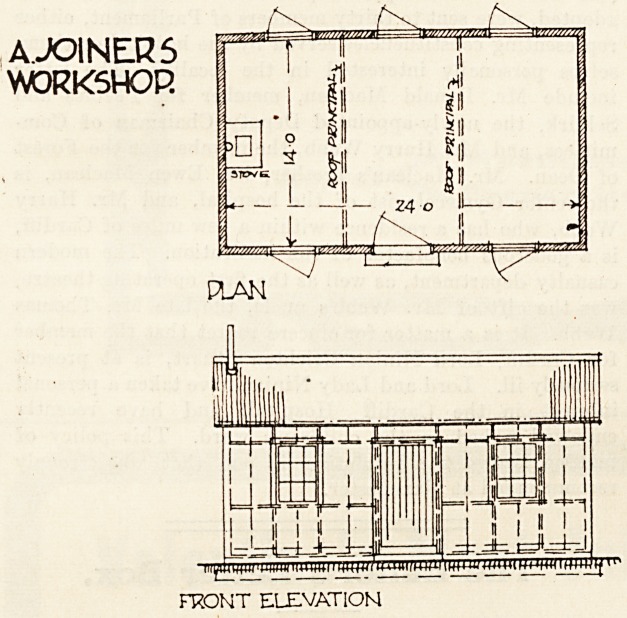


**Figure f2:**